# Anti-inflammatory and/or immunomodulatory activities of *Uncaria tomentosa* (cat’s claw) extracts: A systematic review and meta-analysis of *in vivo* studies

**DOI:** 10.3389/fphar.2024.1378408

**Published:** 2024-05-31

**Authors:** Gustavo Marin Arado, Pedro de Padua G. Amatto, Mozart Marins, Elen Sanchez Rizzi, Suzelei de Castro França, Juliana da Silva Coppede, Fábio Carmona, Ana Maria Soares Pereira

**Affiliations:** ^1^ Department of Biotechnology, University of Ribeirão Preto, Sao Paulo, Brazil; ^2^ Department of Pediatrics, Ribeirao Preto Medical School, University of São Paulo, São Paulo, Brazil

**Keywords:** inflammation, inflammatory diseases, inflammatory mediators, Rubiaceae, medicinal plant

## Abstract

**Background:**

*Uncaria tomentosa* (Willd. ex Schult.) DC. (Rubiaceae) is traditionally used by Amazonian indigenous groups to treat inflammatory diseases. To date, there are no systematic reviews and meta-analyses on the use of *U. tomentosa* for inflammation control in animals supporting the traditional knowledge about this species. This study was conducted to evaluate the effect of *U. tomentosa* extracts in modulating inflammatory mediators and to determine which types of inflammatory diseases can be treated by this species.

**Methods:**

We conducted a systematic review and meta-analysis of preclinical studies published before 26 July 2023, identified in PubMed, Embase, and Scopus. Four independent reviewers extracted the data and assessed the risks of bias. The effects of *U. tomentosa* on inflammatory diseases and the inflammatory mediators involved were extracted from the studies. Standardized mean differences (SMD) and 95% confidence intervals (95%CI) of the outcomes were estimated. The meta-analyses were conducted using RevMan 5.4 (Cochrane Collaboration). This protocol was registered in PROSPERO (CRD42023450869).

**Results:**

Twenty-four of 523 studies were included. *U. tomentosa* extracts decreased the cytokines interleukin (IL)-6 (SMD: −0.72, 95%CI: −1.15, −0.29, *p* = 0.001) and transcription factor nuclear factor *kappa*-B (NF-κB) (SMD: −1.19, 95%CI: −1.89, −0.48, *p* = 0.001). However, the extracts did not significantly alter IL-1 (SMD: −0.16, 95%CI: −0.87, +0.56, *p* = 0.67), IL-10 (SMD: −0.05, 95%CI:–0.35, 0.45, *p* = 0.80), or tumor necrosis factor-*alpha* (TNF-α) levels (SMD: 0.18, 95%CI: −0.25, 0.62, *p* = 0.41).

**Conclusion:**

Many extracts of stem bark, roots, and leaves of *U. tomentosa*, mostly aqueous and hydroethanolic, exhibited anti-inflammatory and/or immunomodulatory activities and low toxicity. The extracts decreased NF-κB and IL-6. These findings suggest that this species has the potential to treat inflammatory diseases in which these markers are increased, according to the ethnopharmacological use. These activities are not related to a specific class of compounds.

**Systematic Review Registration:** https://www.crd.york.ac.uk/prospero/display_record.php?RecordID=450869, Identifier CRD42023450869.

## Highlights

• *Uncaria tomentosa* (cat’s claw) extracts decreased IL-6 and NF-κB.
• Cat’s claw did not alter IL-1, IL-10, or TNF-α.
• Cat’s claw extracts have low toxicity.
• Cat’s claw improves parameters of chronic inflammatory diseases.

## 1 Introduction

*Uncaria tomentosa* (Willd. ex Schult.) DC. (*Rubiaceae*), commonly known as cat’s claw or “unha de gato,” is widely used in Peruvian and Brazilian traditional medicine as an anti-inflammatory, antinociceptive, and antiasthmatic agent, and to prevent diseases (Reinhard, 1999; Valente, 2013; Valdiviezo-Campos et al., 2020; Obregon Vilches, 1997). This species is distributed in Brazil in the states of Acre, Amapá, Amazonas, and Pará (Honório et al., 2016). It is included in the National List of Essential Medicines (Relação Nacional de Medicamentos Essenciais, RENAME), which is provided by the Brazilian Ministry of Health to all municipalities through the National Health System (Sistema Único de Saúde, SUS) (Brazil, 2022).

*U. tomentosa* is rich in alkaloids, including tetracyclic indole, tetracyclic oxindole, pentacyclic indole, pentacyclic oxindole, and glycoindole alkaloids (Laus et al., 1997; Keplinger et al., 1999; Falkiewicz and Lukasiak, 2001). It also contains triterpenoids derived from quinovic acid and polyphenols (Hoyos et al., 2015). Mitraphylline and isopteropodine are considered the chemical markers of this species (USP, 2023). Pharmacological studies using different *U. tomentosa* extracts have confirmed their antiasthmatic, antidiabetic, antimicrobial, anticancer, antioxidant, and anti-inflammatory properties, as well as their neuroprotective effects against Parkinson’s and Alzheimer’s diseases (Sandoval et al., 2002; De Martino et al., 2006; Ciani et al., 2018; Xu et al., 2021; Blanck et al., 2022).

Inflammation is a multifactorial condition that involves several mediators. The latter are potent chemical substances found in the body tissues, such as lymphokines, leukotrienes, prostaglandins, prostacyclins, interferon-*alpha* (IFN-α) and *gamma* (IFN-γ), interleukins (ILs) (Ricciotti and FitzGerald, 2011; Cerami, 1992; Serhan and Levy, 2018), histamine, 5-hydroxytryptamine (5-HT), and tumor necrosis factor-*alpha* (TNF-α) (Holtmann and Neurath, 2004; Branco et al., 2018). It is therefore challenging to find a drug that simultaneously acts on multiple targets and attenuates the damage caused by chronic inflammation (Chen et al., 2018). There is a constant search for plants and substances that are more effective in treating inflammatory diseases by acting on multiple targets with fewer side effects. The anti-inflammatory properties of many substances from medicinal plants have been described (Gandhi et al., 2022a; Gandhi et al., 2022b; Zhao et al., 2023), but their safe clinical use has not yet been proven.

Therefore, the present systematic review aimed to synthesize the knowledge on the preclinical anti-inflammatory and/or immunomodulatory activities of different extracts of *U. tomentosa* evaluated in different *in vivo* models and on their main mechanisms of action, including a meta-analysis of their effects on selected inflammatory mediators.

## 2 Methods

### 2.1 PICOS question and strategy

The review followed the Preferred Reporting Items for Systematic Reviews and Meta-Analyses (PRISMA) guidelines (Page et al., 2021) and was previously registered in PROSPERO (CRD42023450869).

The research questions were as follows: against which inflammatory diseases are *U. tomentosa* extracts effective, as assessed in *in vivo* models, and which inflammatory mediators are involved?

The PICOS strategy (problem, intervention, control, outcomes, and study design) was built as follows: P: inflammatory diseases; I: treatment with *U. tomentosa* extracts; C: no treatment or placebo (vehicle); O: levels of inflammatory mediators; and S: *in vivo* preclinical studies.

### 2.2 Data sources and bibliographic searches

Searches were performed in the PubMed, Scopus, and Embase databases in September 2023 using a combination of keywords, MeSH terms, and their synonyms, as follows: for Embase: (‘inflammation'/exp OR ‘acute inflammation’ OR ‘inflammation’ OR ‘inflammation reaction’ OR ‘inflammation response’ OR ‘inflammatory condition’ OR ‘inflammatory lesion’ OR ‘inflammatory process’ OR ‘inflammatory reaction’ OR ‘inflammatory response’ OR ‘inflammatory syndrome’ OR ‘reaction, inflammation’ OR ‘response, inflammatory’ OR ‘inflammatory disease'/exp OR ‘inflammatory disease’ OR ‘disease, inflammatory’ OR ‘cytokine'/exp OR ‘cytokine’ OR ‘cytokines’ OR ‘interleukin’ OR ‘interferon'/exp OR ‘cl 884′OR ‘cl884′OR ‘endogenous interferon’ OR ‘exogenic interferon’ OR ‘ifn’ OR ‘interferon’ OR ‘interferon 1′OR ‘interferon i' OR ‘interferon type i' OR ‘interferone’ OR ‘interferonogen’ OR ‘interferons’ OR ‘interferron’ OR ‘tumor necrosis factor'/exp OR ‘tnf alfa’ OR ‘tnf alpha’ OR ‘cachectin’ OR ‘cachetin’ OR ‘human recombinant tumour necrosis factor alpha’ OR ‘mhr 24′OR ‘recombinant tumour necrosis factor alpha’ OR ‘tissue necrosis factor’ OR ‘tumor necrosis factor’ OR ‘tumor necrosis factor alfa’ OR ‘tumor necrosis factor alpha’ OR ‘tumor necrosis factor-alpha’ OR ‘tumor necrosis factors’ OR ‘tumor necrosis serum’ OR ‘tumour necrosis factor’ OR ‘tumour necrosis factor alfa’ OR ‘tumour necrosis factor alpha’ OR ‘tumour necrosis factor-alpha’ OR ‘tumour necrosis factors’ OR ‘tumour necrosis serum’) AND (‘uncaria tomentosa'/exp OR ‘uncaria tomentosa’ OR ‘cat`s claw’ OR ‘uncaria tomentosa extract'/exp OR ‘uncaria tomentosa extract').

### 2.3 Study selection and eligibility criteria

Three independent reviewers (GA, PPGA, and AMSP) analyzed the search results and selected potentially relevant studies after reading their titles and abstracts, and using the Rayyan software (Ouzzani et al., 2016). Disagreements were resolved by consensus among the reviewers, with the assistance of a fourth reviewer (JSC), when necessary. The following inclusion criteria were applied: *in vivo* study of inflammatory diseases in animals, administration of *U. tomentosa* extracts *versus* placebo or no treatment, assessment of effects on inflammatory mediators or cytotoxic effects, and published in English, Portuguese, or Spanish. Studies using *U. tomentosa* extracts mixed with other species or isolated substances, non-controlled studies, narrative or scoping review articles, abstracts, conference papers, editorials/letters, and case reports were excluded. Additionally, the reference lists of all selected studies were hand searched to identify additional primary studies for inclusion.

### 2.4 Data extraction

The following data were extracted from the included articles: author, part of the plant used, type of extract (solvent, method, and extraction time), concentration and/or dose, animal species, results (cytotoxicity and inflammatory mediators), and conclusions. Treatment effects for continuous outcomes were extracted as mean differences (MD) plus standard deviations (SD), which could also be estimated from standard errors or confidence intervals. In the studies where such values were not reported, they were estimated from charts using ImageJ software (National Institutes of Health, Bethesda, USA). The authors of the included studies were contacted when necessary (when some data or articles were not available).

### 2.5 Quality assessment

For the risk of bias, two investigators (AMSP and FC) independently reviewed the selected studies according to a modified CAMARADES checklist (Macleod et al., 2004) and reported the risks of bias in a table. After the initial analysis, the authors reassessed the articles analyzed previously by each other. Any discrepancies were resolved by a third author (GMA) after discussion with the team. The information is presented as a risk of bias summary (Table 2).

### 2.6 Statistical analysis

Review Manager 5.4 (Nordic Cochrane Centre, The Cochrane Collaboration, Copenhagen, Denmark) was used for statistical analysis. Heterogeneity was evaluated using Cochrane’s Q test and I^2^ statistics, with a *p*-value <0.10 and I^2^ > 50% being considered significant, respectively. We built a fixed-effects model for endpoints with I^2^ < 50% (low heterogeneity). In the case of pooled outcomes with high heterogeneity, a random-effects model was applied. The results were reported as standardized mean differences (SMD) and their respective 95% confidence intervals (95%CI). We also performed a subgroup analysis to assess the effects of different extracts (aqueous, hydroethanolic, and ethanolic) on the outcomes.

## 3 Results

### 3.1 Study selection and characteristics

**FIGURE 1:**
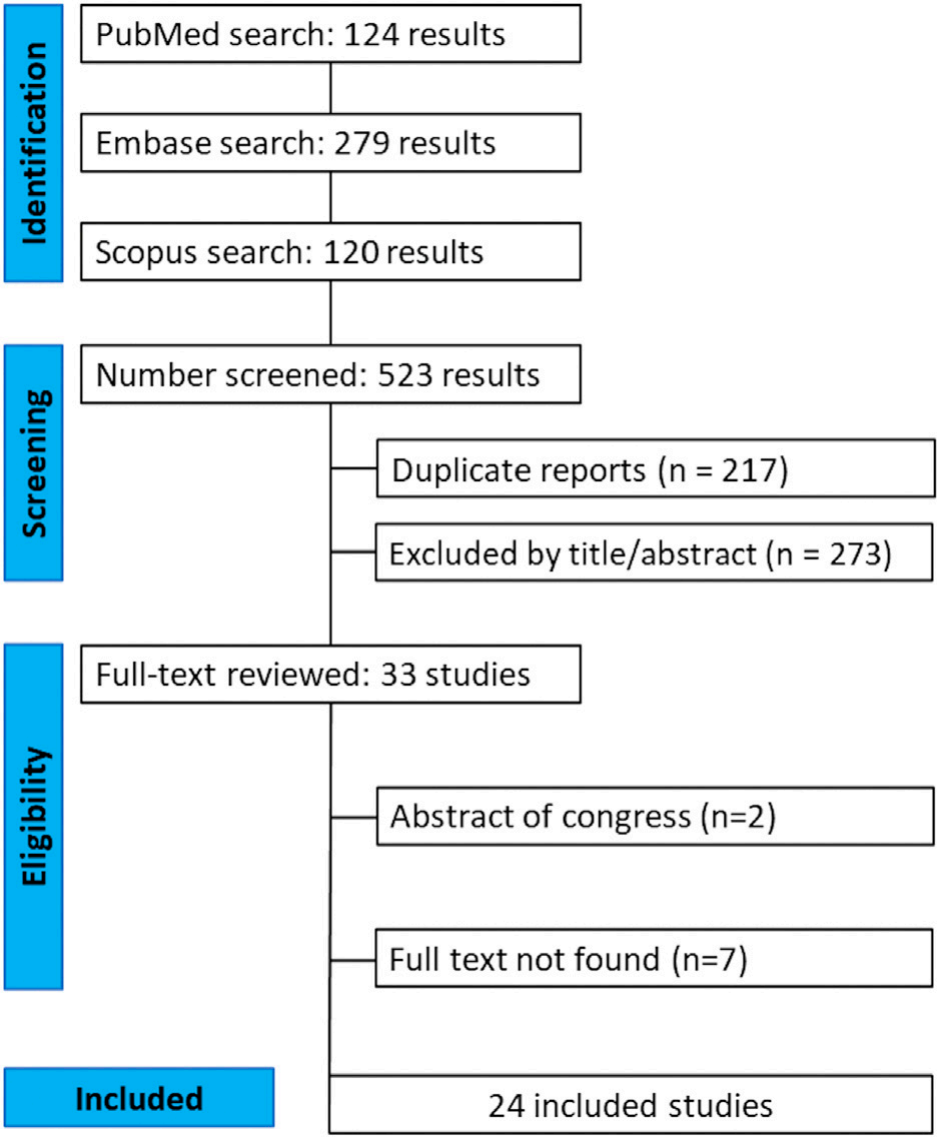
PRISMA flow diagram of study screening and selection.

The initial search retrieved 523 studies published between 1989 and 2023; of these, 124 were found in PubMed, 279 in Embase, and 120 in Scopus. There were 217 duplicated articles, and 273 were excluded after reading the title and the abstract. Thus, 33 studies were selected for full-text reading. Two additional articles were later excluded because they were conference abstracts, whereas seven could not be obtained in full. The final number of articles was 24 (Figure 1).

Research workers from eleven countries across the American, European, and Asian continents have published preclinical studies with extracts of *U. tomentosa*, demonstrating the scope of interest and use of this plant in the world. The countries that have contributed most to the publications of *in vivo* studies are Brazil (41.6%), the United States (12.5%), and Peru (12.5%).

The results of the included studies show that *U. tomentosa* extracts are well tolerated by the animals and have little or no toxicity at the doses evaluated (Table 1).

**TABLE 1:** Characteristics of the included studies regarding plant part, type of extract, disease model, treatment, and main results.

Plant part, extract preparation, and chemical composition	Model/experimental methods	Main results	Authors’ conclusion	Authors, year (country)
Stem bark	Carrageenan-induced paw edema in male Wistar rats. Doses: aqueous: 84 mg/kg chloroform–methanol 50 mg/kg. Route: gavage. Frequency and duration: administered 1 h before edema induction and hourly for 5 h	Petroleum ether, chloroform, and methanol extracts were not active. Chloroform–methanol and aqueous extracts were active, displaying 69.2% and 41.2% inhibition of the maximum edema (3 h), respectively. The compound quinovic acid-3-ß-*O*-(ß-D-quinovopyranosyl)-(27|–> 1)- ß -D-ghcopyranosyl ester caused 33% inhibition at 3 h	Extracts of medium and high polarity reduced edema in the paws of male Wistar rats, and quinovic acid glycoside was found to be an anti-inflammatory compound	Aquino et al., 1991 (Italy)
400 g was extracted with petroleum ether, chloroform, chloroform–methanol (9:1), methanol, and water. Chemical composition of the chloroform–methanol extract: 5-α carboxystrictosidine, oleanolic acid, ursolic acid, 3ß, 6ß, 19α trihydroxyurs-12-en-28-oic acid, 23-oxo and 23-nor-24-esomethylene and quinovic acid-3-ß-*O*-(ß-D-quinovopyranosyl)-(27|–> 1)-ß-D-glicopyranosyl				
Stem bark	Models of lipid peroxidation and oxidative damage to DNA. Dose: 300 mg/kg.	UT reduced the production of thiobarbituric acid-reactive substance production (TBARS)	The anti-inflammatory activity of UT may be related, at least in part, to its ability to suppress lipid peroxidation that occurs during the inflammatory response, as well as to polyphenolic compounds of plant	Desmarchelier et al., 1997 (Brazil and Argentina)
10 g of dry bark was extracted with 100 mL of methanol for 24 h and then concentrated in a speed vac	Route: subcutaneous. Frequency and duration: the dose was administered once, and after 4 h, the animals were killed			
Stem bark	Indomethacin-induced chronic intestinal inflammation. Dose: 5 mg/mL.	Inflammation, mucosal ulceration, and nodules in the intestine were reduced by the extract, and there was a significant reduction in myeloperoxidase activity	The aqueous extract decreased indomethacin-induced intestinal inflammation and reduced the activity of myeloperoxidase in rats	Sandoval-Chacón et al., 1998 (the United States)
An aqueous decoction was prepared with 20 g/L for 30 min.	Route: oral.			
The chemical composition is not reported	Frequency and duration: *ad libitum* in drinking water (5 mg extract/mL water) for 7 days			
Stem bark	Carrageenan-induced mouse paw edema. Doses: 500, 200, 100, and 50 mg/kg. Route: gavage. Frequency and duration: frequency was not clearly reported, dose(s) was administered for 8 days, and outcomes were assessed 4 h after carrageenan injection	Hydroalcoholic (50, 100, 200, and 500 mg/kg) and aqueous (200 mg/kg) extracts inhibited paw edema with effects like indomethacin (7 mg/kg)	The hydroalcoholic extract showed dose-dependent anti-inflammatory action. Although the hydroalcoholic extract was more efficient than the aqueous extract, these extracts were not obtained from the same plant material, so the difference in alkaloid content cannot be attributed to the extraction procedure	Aguilar et al., 2002 (Peru and Germany)
Hydroalcoholic (80% ethanol) spray-dried extract				
(drug extract ratio: 8:1)				
Chemical composition: 5.61% of total oxindole alkaloids				
Aqueous freeze-dried				
Extract				
Chemical composition: 0.26% oxindole alkaloids				
Stem bark	Indomethacin-induced acute gastric injury in male Sprague–Dawley rats. Dose for scavenging effect on DPPH radical: 1, 3, 10, 30, and 100 μg/L. Dose for LPS-mediated nitric oxide production and TNFα production by macrophages: 100 ng/mL. Dose for scavenging effect on ABTS-radicals: 10 µL of UT dissolved with 990 µL water. Dose for gastric injury induced by indomethacin: 5 mg/mL in drinking water. Route: oral. Frequency and duration: 3 days prior to the administration of indomethacin	UT showed cytoprotective effects on stomach epithelial cells of rats exposed to indomethacin and blocked the expression of TNF-α	UT’s anti-inflammatory and antioxidant activities are independent of oxindole or pentacyclic alkaloid content	Sandoval et al., 2002 (the United States and Peru)
The extract of the bark was prepared by decoction using boiling water (50 g/L w/v) for 30 min. Alkaloids present in the extract (mg/g): speciophylline (1.60), mitraphylline (0.88), uncarine F (2.02), pteropodine (0.15), isomitraphylline (1.67), and uncarine E (2.72)				
Stem bark	Ozone-induced (8 h) pulmonary inflammation. Doses: 50% (in distilled water) and 100% decoction.	Treated mice (higher dose) showed lower protein levels in bronchoalveolar lavage fluid, a lower degree of epithelial necrosis, a greater number of intact epithelial cell nuclei in the bronchial wall, a reduction in the number of infiltrating neutrophils in the bronchial lumen, and a reduction in PMNs per unit length of bronchial epithelium	UT extract appeared to prevent O_3_-induced pulmonary inflammation in male mice	Cisneros et al., 2005 (the United States)
An aqueous decoction was prepared (20 g/L) for 3 h, and the yield was 14 mL of aqueous extract per gram of dry bark. Chemical composition: not reported	Route: oral. Frequency and duration: *ad libitum* for 8 days			
Stem bark	Male BALB/c mouse model of listeriosis. Doses: 10, 50, 100, 150, and 200 mg/kg. Route: oral. Frequency and duration: 7 days prior to infection	Treatment with 100 mg/kg of UT decreased levels of IL-1 at 72 h and maintained elevated levels of IL-6 throughout the experiment. The dose of 100 mg/kg upregulated the production of colony-stimulating factors (CSFs)	UT indirectly modulates immune activity by inducing a greater reserve of myeloid progenitors in the bone marrow as a result of the release of biologically active cytokines (CSF, IL-1, and IL-6)	Eberlin et al., 2005 (Brazil)
Dry extract (total alkaloid content of 1%) by Galena Química e Farmaceutica Ltda (Campinas, SP, Brazil)				
Stem bark	LPS-induced inflammatory response, tumor growth, and metastasis in the B16-BL6 melanoma model in C57BL/6 mice. Doses: LPS: 50 μg/animal daily for 3 days before the LPS challenge. LPS in tumor-bearing mice: protocol a) 50 µg/animal on days −2, −1, and 0; and protocol b) the same dose of extract 5 times per week starting from day 0 up to day 21.	UT reduced IL-6 and NO by 35% and 62%, respectively	UT showed an inhibitory effect on pro-inflammatory cytokine production *in vivo* and inhibited tumor growth and metastasis in the same mouse model	Fazio et al., 2008 (Venezuela)
Ground material was macerated in a 70% (ethanol:water) solution for 21 days. A stock solution was prepared at a concentration of 5 mg/mL, calculated from the dry weight of a lyophilized sample	Tumor growth and metastasis: 50 μg/animal daily for 3 days prior to inoculation. Route: intraperitoneal. Dose for the cells: 10, 30, and 100 μg/mL for 1 h	TNF-α and IL-6 in primary tumor animals were significantly reduced by UT (85% and 81%, respectively). UT inhibited tumor growth and metastasis *in vivo*		
Stem bark	Spleen cell chemokinesis (spontaneous migration) in female BALB/c mice. Doses: 200 and 2000 μg/mouse. Route: oral. Frequency and duration: daily for 7 days	The dose of 200 µg showed high splenocyte *in vitro* migratory activity, whereas the 2000 μg dose provided a less stimulating effect	Both doses of extract stimulated splenocyte mobility, but the lower dose of extract was most effective	Nowakowska et al., 2009 (Poland)
The aqueous extract was prepared with 10 g of powdered bark in 100 mL of water. Chemical composition: fractionation was patented, and the alkaloid content was 0.43%, with a predominance of uncarine C and isomitraphylline				
Stem bark	LPS-induced pulmonary injury in Swiss albino mice	The extract did not cause any changes in weight nor did it change the structure of the kidneys, liver, or lungs. There was a reduction in infiltrate, congestion, pulmonary edema, and the number of neutrophils and macrophages in the bronchoalveolar fluid	The extract was safe at the doses used. It also showed anti-inflammatory effects in mice with lung disease in the model studied	Roque et al., 2009 (Argentina)
The extract was freshly prepared by decoction of the bark at a 20 g/L concentration for 45 min, then filtered and left for 12 h before use. Chemical composition: not reported	Dose: 0.75 g/kg. Route: oral. Frequency and duration: *ad libitum* for 7, 15, 30, and 90 days			
Stem bark	Immunosuppression is induced by ifosfamide in male BALB/c mice. Doses: 5 and 15 mg/animal.	UT reversed ifosfamide-induced neutropenia, increasing the neutrophil count 4-fold (5 mg) and 13-fold (15 mg) compared to the control group, an effect like filgrastim. There were no differences in the levels of non-protein thiols or in the activities of the antioxidant enzymes catalase or superoxide dismutase	At the doses tested, UT reversed ifosfamide-induced neutropenia	Farias et al., 2011 (Brazil)
The extract was prepared by ultra-turrax extraction (Biotron-Kinematica AG) with 70% ethanol. The alcohol was removed by spray drying (Centroflora). Chemical composition: the extract has a content of 2.57% of pentacyclic oxindole alkaloids (speciophylline–0.26%; uncarine F–0.07%; mitraphylline–0.80%; isomitraphylline–0.40%; uncarine C–0.46%; and uncarine E–0.58%)	Route: oral. Frequency and duration: daily for 4 days			
Stem bark	*In vivo* and *in vitro* immunotoxicity and immunomodulatory effects. Doses: *in vivo*: 125, 500, and 1,250 mg/kg; *in vitro*: 10–500 μg/mL. Route: oral. Frequency and duration: *in vivo*: daily for 28 days; *in vitro*: 48 h	UT did not change the body weight of the animals, but it did increase the weight of the liver, spleen, and kidneys. The extract was not toxic to lymphocytes and increased cell viability. There was an increase in the production of IL-4 and IL-5 and a strong inhibition of IFN-γ and IL-2. The levels of TNF-α were only increased at a dose of 100 μg/mL. The extract increased the concentration of CD4^+^ T lymphocytes	The extract showed low systemic toxicity, was not immunotoxic, and was able to modulate distinct patterns of the immune system in a dose-dependent manner. UT showed immunomodulatory activity and promoted a cytokine bias towards a Th2 profile, suggesting its potential to treat Th1 immune-mediated disorders	Domingues et al., 2011a) (Brazil)
Extracted in water:ethanol 1:1. The crude hydroalcoholic extract was extracted with 0.1 N HCl and then partitioned with ethyl acetate. The aqueous fraction was treated with NH_4_OH until a pH of 9–10 was reached, and then the mixture was extracted with ethyl acetate		The 1,250 mg/kg dose increased the concentration of CD45RA + B lymphocytes		
Chemical composition: 1% of total alkaloids				
Stem bark Hydroethanolic extract (1:1). The crude hydroalcoholic extract was extracted with 0.1 N HCl and then partitioned with ethyl acetate. The aqueous fraction was treated with NH_4_OH until a pH of 9–10 was reached, and then the mixture was extracted with ethyl acetate (1% of total alkaloids)	Immune-mediated diabetes induced by multiple low-doses of streptozotocin (MLDS).	All doses produced a significant reduction in blood glucose levels and the incidence of diabetes at the end of the experiment. The 400 mg/kg dose completely reversed diabetes on day 12. Structural differences in the spleen, lymph nodes, kidneys, thymus, femur, and liver were not induced by UT. UT produced significant protection against mononuclear infiltration, characterized by a higher percentage of intact islets and a concomitant reduction in the percentage of moderate, severe, and destructive insulitis. UT normalized the percentage of T CD4^+^ and Treg CD4^+^CD25+Foxp3+ lymphocytes. UT prevented the increase in IFN-γ and increased the production of IL-4 and IL-5 (Th2 polarization)	UT protected mice against immune-mediated diabetes through Th2 polarization, normalization of T CD4^+^ and Treg CD4^+^CD25+Foxp3+ lymphocytes, or both mechanisms	Domingues et al., 2011 b) (Brazil)
	Doses: 10, 50, 100, or 400 mg/kg.			
	Route: gavage.			
	Frequency and duration: daily for 21 days			
Stem bark	Healthy Wistar Hannover rats. Doses: 15, 75, and 150 mg/kg. Route: gavage. Frequency and duration: daily for 90 consecutive days	Animals receiving 75 mg/kg had increased alanine transaminase (ALT), whereas those receiving 75 or 100 mg/kg had decreased blood glucose levels. Only rats treated with 75 mg/kg of UT showed immunomodulatory effects on macrophage activity.	UT decreased blood glucose levels and should be used with caution by diabetic and non-diabetic patients	Mendes et al., 2014 (Brazil)
The dried extract was provided by the manufacturer: “Santosflora: Herbs, Spices and Dry Extracts, Ltd.” (Tatuape, Sao Paulo, Brazil), and it contained 0.65% of total alkaloids. Chemical composition: pteropodine (20%), isopteropodine (12%), isomytraphylline (10%), speciophylline (11%), uncarine F (4%), and mytraphylline (18%). The content of tetracyclic alkaloids was determined at 25% as follows: ryncophilline (15%) and isoryncophylline (10%). Corynoxeine and isocorynoxeine are considered trace alkaloids		Long-term administration did not alter blood counts		
Stem bark	B16 melanoma in C57BL/6 mice. Doses: 50, 100, 500, and 1,000 mg/kg. Route: gavage. Frequency and duration: one dose 7 days before inoculation of B16 cells and daily for 22 days after inoculation	UT increased the CD4/CD8a ratio (1,000 mg/kg) and the CD4^+^CD44+/CD8a+CD44^+^ ratio (50 and 500 mg/kg). It increased the proportion of myeloid dendritic cells. It induced a pro-inflammatory Th1 profile and reduced the Th17 response. It increased TNF-α and reduced IL-17A and IL-2, whereas IL-4 was not altered. Within the tumor: there were no differences in IL-12p70 and MCP-1	UT shows immunomodulatory effects that are better systemic than intratumoral	Lozada-Requena et al., 2015 (Peru)
The dry hydroalcoholic extract was prepared by decoction with ethanol and water in a ratio of 70:30 for 1 h at 20 °C (Peruvian Heritage^®^). Chemical composition: 5.03% pentacyclic oxindole alkaloids				
Root bark	Adjuvant-induced arthritis model in Wistar rats. Dose: 150 mg/kg.Route: gavage. Frequency and duration: twice daily for 45 days	UT partially reduced mechanical sensitivity, paw thickness, and MPO activity, and prevented an increase in E-NTPDase activity in lymphocytes. UT reduced serum levels of ATP and increased ADP in arthritic animals	The extract of UT had an effect on arthritis, and purinergic signaling is involved in these responses	Castilhos et al., 2015 (Brazil)
Commercially available dry extract (5.0 mg of total alkaloids, expressed as mitraphylline) (Herbarium Botanical Laboratory, PR-Brazil)				
Root bark	Models of obesity: high-fat diet (HFD) and genetically obese (ob/ob) mice. Doses: 50 mg/kg. Route: gavage. Frequency and duration: daily for 5 days	UT improved fasting blood glucose and insulin sensitivity and reduced liver inflammation. It reduced body mass index and increased energy expenditure in obese mice. It reduced fasting blood glucose levels, improved blood glucose homeostasis, and improved liver insulin signaling in obese mice. It reduced liver inflammation. UT induced an intracellular reduction in the expression levels of JNK, Ikkβ, NF-kB, and TNF-α. It reduced F4/80 mRNA levels and reversed increased IL-1β expression. It induced IL-10 and arginase 1 expression. UT reduced the number of F4/80-positive cells in the liver	UT decreased non-alcoholic fatty liver disease and liver inflammation, and improved insulin sensitivity	Araujo et al., 2018 (Brazil)
Commercially available dry extract (5.0 mg of total alkaloids, expressed as mitraphylline) (Herbarium Botanical				
Laboratory, PR-Brazil)				
Contains uncarine D, uncarine F, and mitraphylline (major compounds), in addition to isomitraphylline, uncarine C, and uncarine E				
Stem bark	Murine model of ovalbumin-induced asthma. Doses: 50, 100, and 300 mg/kg. Route: intraperitoneal. Frequency and duration: daily for 7 days	Bark and leaf extracts did not alter TNF-α production but inhibited the transcription of NF-kB. The extracts decreased the total number of inflammatory cells in bronchoalveolar lavage fluid (BAL) samples. Bark and leaf extracts decreased eosinophilia in BAL, but bark extract was more effective. The 200 mg/kg dose of bark extract killed all animals. The extracts did not alter TNF-α. The extracts reduced eosinophils and increased neutrophils. Macrophages and lymphocytes were not affected. The extracts increased TGF-β and did not alter IgE levels	UT shows anti-inflammatory activity in an asthma model by inhibiting pro-inflammatory cytokines. The bark extract was more effective on asthmatic inflammation, whereas the leaf extract was more effective in controlling respiratory mechanics	Azevedo et al., 2018 (Brazil)
An aqueous decoction of 20 g/L stem bark was prepared by boiling it in water for 5 min, yielding 1.2 g of lyophilized dry extract (6.6 μg/mL of mitraphylline)				
Leaves				
The same procedure was carried out with the leaves, but by infusion, yielding 1.0 g of dry extract (14.7 μg/mL of mitraphylline)				
Plant parts were not reported. Plant extract (10:1) with 1.5% oxindole alkaloids (Biotanica Co., New Zealand)	Liver damage in male Wistar rats exposed to fipronil. Dose: 120 mg/kg. Route: gavage. Frequency and duration: daily for 6 weeks	UT increased body weight, reduced liver weight, and controlled ALP, ALT, and AST elevations. UT kept TNF-α and IL-6 synthesis unchanged. It reversed the increase in oxidative stress. UT attenuated NF-kB activity in the liver. UT reduced hepatic inflammation and necrosis, in addition to preserving the normal hepatic morphological architecture	UT reduced oxidative stress and liver injury induced by fipronil *via* NF-kB inhibition	Elgawish et al., 2019 (Egypt)
Leaves	Resistance to oxidative stress in *Caenorhabditis elegans.* Doses: 10, 20, 40, 80, and 100 μg/mL. Route: intraperitoneal. Frequency and duration: for 48 h at 20 °C	UT showed moderate antioxidant activity when compared to ascorbic acid. UT at 40 μg/mL significantly reduced the intracellular accumulation of ROS by 25.58%. The survival rate of jugloneurine stressed worms pretreated with 80 μg/mL UT was 53.91%, which was comparable to that of worms pretreated with 50 μg/mL EGCG (58.84%). At 80 μg/mL UT, SOD-3 expression was reduced by 12.08% compared to the untreated control of 23.82%	UT reduced ROS levels in worms lacking the DAF-16 transcription factor. This activity in *C. elegans* is independent of its alkaloid content and is probably mediated by different stress signaling pathways	Azevedo et al., 2019 (Brazil)
The aqueous extract of the leaves (20 g/L) was prepared by infusion and left for 30 min, yielding 1.3 g of dry extract containing mitraphylline, isomitraphylline, and isorhynchophylline				
Stem bark	Fipronil-induced liver toxicity in male Wistar rats	Administration of UT extract significantly reduced neutrophils and increased lymphocytes and monocytes in the blood. The percentage of DNA in the tail, the length of the tail, and the percentage of DNA damage were significantly reduced in rats treated with UT. UT reversed the oxidative stress. Animals receiving UT showed normal splenic architecture	UT ameliorated fipronil-induced oxidative damage and immunotoxicity, as well as endocrine disruption. These effects are probably related to the flavonoid and phenolic compounds	Aldayel et al., 2021 (Egypt)
Extract containing 3% oxindole alkaloids as indicated by the manufacturer and chlorogenic acid μg/g (major compound) (Maple Lifesciences, India.)	Dose: 120 mg/kg. Route: oral. Frequency and duration: daily for 30 days			
Stem	Streptozotocin-induced Alzheimer disease modeled in adult male Sprague–Dawley rats. Dose: 400 mg/kg. Route: gavage. Frequency and duration: daily for 6 weeks	The rats treated with UT took less time to reach the platform, and the STZ-treated rats that received UT spent significantly more time in the target quadrant than vehicle-treated rats. UT treatment inhibited the hyperphosphorylation of tau protein at the sites of S396 and S404. UT suppressed the elevated levels of IL-1β, IL-6, and TNF-α. UT exerted antioxidant effects by increasing the activities of antioxidant enzymes (superoxide dismutase, catalase, and glutathione peroxidase) and the expression of the HO-1 protein	UT ameliorated cognitive impairments in SZT-induced Alzheimer’s disease rats. This activity has been attributed in part to the protective effects of the alkaloids rhynchophylline and isorhynchophylline	XU et al., 2021 (China)
In this, 1,000 g of stem was macerated in 10 L of 70% ethanol:water for 24 h at room temperature and then extracted in an ultrasonic bath for 1 h. The material was freeze-dried. The yield of the extracts was 13.88%. The contents of alkaloids were as follows: rhynchophylline (0.278%), isorhynchophylline (0.531%), corynoxeine (0.010%), and isocorynoxeine (0.028%)				
Stem bark	Angiotensin (Ang) II-induced pregnancy hypertension *in vivo*. Dose: 4 mg/mL in drinking water. Frequency and duration: *ad libitum* for 3 weeks. Route: oral *In vitro*. Dose: 100 or 200 μg/mL for 24 h	Treatment with AC-11 decreased AngII-induced hypertension. AC-11 decreased the population of CD8+T cells, the ratio of CD8/CD4, and plasma interleukin-6 levels in pregnant and non-pregnant mice. AC-11 decreased plasma levels of sFlt-1 and sEng in pregnant mice, and this effect was confirmed in an *in vitro* assay	AC-11 has the potential to control hypertension in non-pregnant and pregnant patients by balancing T-cell populations and inhibiting factors associated with hypertensive disorders of pregnancy	Oogaki et al., 2021 (Japan)
Extract AC-11^®^ from Optigenex Inc. (Scottsdale, AZ, USA).				
Extract containing 10% carboxy alkyl esters				
Leaves	Murine model of ovalbumin-induced asthma. Dose: 200 μg/animal. Route: intraperitoneal. Frequency and duration: daily for 7 days	UT reduced OVA-induced histopathological inflammation by 62.17%, reduced bronchial hyperplasia by 48.68%, and decreased edema and vascular changes by 56.39%	UT has anti-asthmatic activity comparable to methylprednisolone in mice	Saghir et al., 2023 (Pakistan)
100 g of ground leaves were extracted in 1 L of ethanol for 24 h at a temperature of 37 °C, and after evaporation, the dry extract was resuspended in ethanol (1 L)				

### 3.2 Meta-analysis of selected inflammatory mediators

#### 3.2.1 Interleukin-1 (IL-1)

**FIGURE 2:**
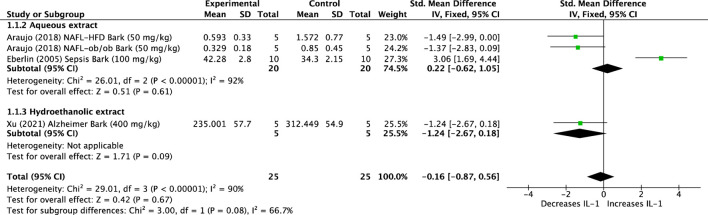
Forest plot of the efficacy of *Uncaria tomentosa* extracts on levels of interleukin (IL)-1.

Three studies that used four animal models and aqueous and hydroethanolic extracts of *U. tomentosa* were analyzed. The results showed no effect on IL-1 levels (SMD: −0.16, 95%CI: −0.87, +0.56, *p* = 0.67). The overall assessment of the data revealed high heterogeneity (*p* < 0.001, I^2^ = 90%) (Figure 2).

#### 3.2.2 Interleukin-6 (IL-6)

**FIGURE 3:**
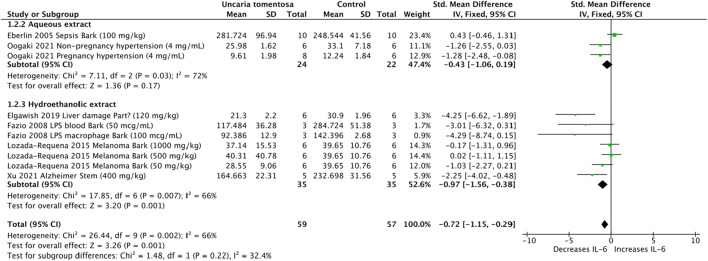
Forest plot of the efficacy of *Uncaria tomentosa* extracts on levels of interleukin (IL)-6.

Six studies showed that the aqueous extract tended to increase IL-6, whereas hydroethanolic extracts significantly reduced IL-6 levels (SMD: −0.72, 95% CI: −1.15, −0.29, *p* = 0.001). The overall assessment of the data showed high heterogeneity (*p* = 0.002, I^2^ = 66%) (Figure 3).

#### 3.2.3 Interleukin-10 (IL-10)

**FIGURE 4:**
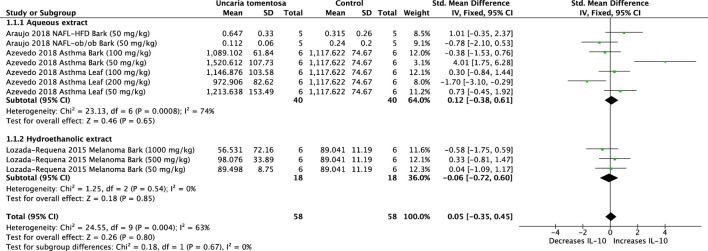
Forest plot of the efficacy of *Uncaria tomentosa* extracts on levels of interleukin (IL)-10.

The effect of *U. tomentosa* on IL-10 was analyzed in three studies. The results showed that the extracts did not significantly alter IL-10 levels (SMD: −0.05, 95%CI:–0.35, 0.45, *p* = 0.80). The overall assessment of the data revealed significant heterogeneity (*p* = 0.004, I^2^ = 63%) (Figure 4).

#### 3.2.4 Tumor necrosis factor-*alpha* (TNF-α)

**FIGURE 5:**
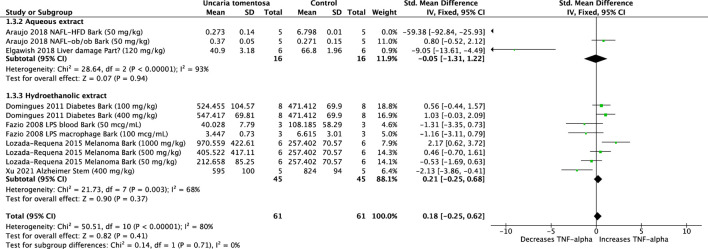
Forest plot of the efficacy of *Uncaria tomentosa* extracts on levels of tumor necrosis factor (TNF)-α.

Six studies using aqueous and hydroethanolic extracts of *U. tomentosa* were analyzed. The extracts did not significantly alter TNF-α levels (SMD: 0.18, 95%CI: −0.25, 0.62, *p* = 0.41). Overall evaluation of the data showed high heterogeneity (*p* < 0.00001, I^2^ = 80%) (Figure 5).

#### 3.2.5 Nuclear factor-*kappa*-B (NF-κB)

**FIGURE 6:**
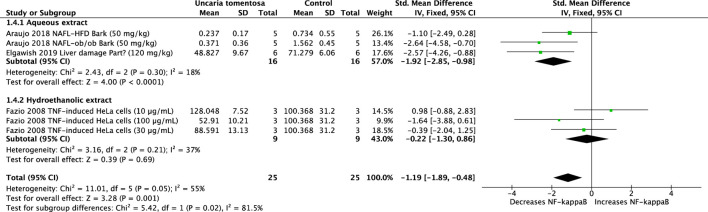
Forest plot of the efficacy of *Uncaria tomentosa* extracts on levels of tumor necrosis factor kappa B (NF-κB).

Four studies using aqueous and hydroethanolic *U. tomentosa* extracts showed a significant reduction in NF-κB (SMD: −1.19, 95%CI: −1.89, −0.48, *p* = 0.001). Overall evaluation of the data revealed moderate heterogeneity (*p* = 0.05, I^2^ = 55%) (Figure 6).

### 3.3 Quality assessment

There was a high risk of bias regarding sample size calculation, missing outcome data (excluded animals), and possible conflicts of interest, and a moderate risk of bias for temperature control, the number of animals appropriate to the model, and compliance with animal welfare regulations. The risk of bias was low for peer reviewing and the disease model (Table 2). Publication bias was high for IL-6 as the funnel plot is shifted toward the left (data not shown).

**TABLE 2:** Risk of bias among the included studies.

	Criteria							
Authors	Publication in a peer-reviewed journal	Control of temperature	Animal model with only inflammation	Sample size calculation	Number of animals appropriate to the model*	Compliance with animal welfare regulations	Bias due to missing outcome data	Statement regarding possible conflict of interest
Aquino et al. (1991)	Y	NM	Y	NM	N	NM	NM	NM
Desmarchelier et al. (1997)	Y	NM	Y	NM	NM	NM	NM	NM
Sandoval-Chacón et al. (1998)	Y	Y	Y	NM	NM	NM	NM	NM
Aguilar et al. (2002)	Y	NM	Y	NM	NM	NM	NM	NM
Sandoval et al. (2002)	Y	NM	Y	Y	NM	NM	NM	NM
Cisneros et al. (2005)	Y	Y	Y	NM	Y	Y	NM	NM
Eberlin et al. (2005)	Y	Y	Y	NM	NM	Y	NM	NM
Fazio et al. (2008)	Y	NM	Y	NM	NM	Y	NM	NM
Nowakowska et al. (2009)	Y	NM	Y	NM	Y	Y	NM	NM
Roque et al. (2009)	Y	Y	Y	NM	Y	Y	NM	NM
Farias et al. (2011)	Y	Y	Y	NM	Y	Y	NM	NM
Domingues et al. (2011)	Y	Y	Y	NM	NM	Y	NM	NM
Domingues et al. (2011)	Y	Y	Y	NM	Y	Y	NM	Y
Lozada-Requena et al. (2015)	Y	NM	Y	NM	Y	Y	NM	Y
Castilhos et al. (2015)	Y	Y	Y	NM	Y	Y	NM	Y
Araujo et al. (2018)	Y	Y	Y	NM	Y	Y	NM	Y
Azevedo et al. (2018)	Y	NM	Y	NM	Y	Y	NM	NM
Elgawish et al. (2019)	Y	Y	Y	NM	Y	Y	NM	Y
Azevedo et al. (2019)	Y	Y	Y	NM	Y	Y	NM	Y
Aldayel et al. (2021)	Y	Y	Y	NM	Y	Y	NM	Y
XU et al. (2021)	Y	Y	Y	NM	Y	Y	NM	Y
Saghir et al. (2023)	Y	Y	Y	Y	Y	NM	NM	Y
Total score (out of 22)	21	14	22	2	14	16	0	9

Legend: Y, yes; NM, not mentioned.

## 4 Discussion

In this systematic review and meta-analysis, *U. tomentosa* decreased the levels of IL-6 and NF-κB but not of IL-1, IL-10, or TNF-α, in animal models of inflammatory diseases. These models included asthma, diabetes, arthritis, obesity, gastric ulcers, and intestinal diseases (Azevedo et al., 2018; Mendes et al., 2014; Castilhos et al., 2015; Araujo et al., 2018; Sandoval et al., 200; Sandoval-Chacón et al., 1998). Although some ethnic groups use this species for religious purposes only, such as the Asháninkas (Keplinger et al., 1999), our findings confirm the anti-inflammatory and/or immunomodulatory activities of the species, as advocated by other indigenous groups of the Amazon.

Inflammatory diseases are usually accompanied by a significant production of reactive oxygen and nitrogen species, as well as the expression of pro-inflammatory cytokines, most notably IL-1, IL-6, and TNF-α; the anti-inflammatory cytokine IL-10; and the activation of NF-κB (Hata et al., 2004; Kany et al., 2019). The studies using extracts of *U. tomentosa* included in this review show decreases in levels of IL-6. These findings explain, at least in part, the anti-inflammatory activity of this species. Most of the *in vivo* studies have demonstrated, directly or indirectly, that the inhibition of the activity of the transcription factor NF-κB by the extracts was accompanied by decreased levels of IL-1 or IL-6 (Aguilar et al., 2002; Sandoval et al., 2002; Sandoval et al., 2002; Lozada-Requena et al., 2015; Araujo et al., 2018; Azevedo et al., 2018; Elgawish et al., 2019; XU et al., 2021). Targeting IL-6 is an important strategy to treat inflammatory diseases. Therapeutic monoclonal antibodies against cytokines or their receptors, such as tocilizumab (Ohsugi, 2020), are among the most effective, yet very expensive, therapies.

In general, *U. tomentosa* extracts have an antioxidant activity that always potentiates their anti-inflammatory activity in the models tested (Sandoval-Chacón., 1998: XU et al., 2021; Desmarchelier et al., 1997; Azevedo et al., 2019; Saghir et al., 2023). Selective inhibitory activity against cyclooxygenase-2 (COX-2) has also been observed, confirming anti-inflammatory properties (Aguilar et al., 2002).

Another important aspect to be considered is that *U. tomentosa* extracts preserve CD4^+^ and CD8^+^ T cells and possibly stimulate cytokines that favor the polarization of CD4^+^ Th2 cells. These cells play an important role in autoimmune diseases, such as rheumatoid arthritis, by modulating the excessive activity of Th1 cells (Domingues et al., 2011a; Domingues et al., 2011b; Lozada-Requena et al., 2015; Azevedo et al., 2018). In fact, an extract of *U. tomentosa* was superior to placebo in 50 patients with rheumatoid arthritis, decreasing the number of painful joints, morning stiffness, pain intensity, and joint edema (Mur et al., 2002). Therefore, *U. tomentosa* should be more broadly investigated for the treatment of autoimmune diseases.

Interestingly, *U. tomentosa* extracts can either stimulate or inhibit the release of different cytokines, depending on the animal’s health status or the modeled disease. This was observed for IL-1: in the study by Eberlin et al. (2005), a sepsis model, *U. tomentosa* increased IL-1 levels throughout the infection; in the study by Araujo et al. (2018), a non-alcoholic fatty liver disease model, extracts of *U. tomentosa* decreased IL-1 levels. Some authors refer to this regulatory effect as immunomodulation (Domingues et al., 2011a; Domingues et al., 2011b; Lozana-Requena et al., 2015; Elgawish et al., 2019; Aldayel et al., 2021; Xu et al., 2021). In our study, *U. tomentosa* did not alter IL-10 levels in a sepsis model, suggesting immunomodulatory activity in infectious diseases.

Regarding the effectiveness of pentacyclic oxindole alkaloids in reducing inflammatory processes, the results are conflicting. Some authors claim that ethanolic extracts enriched with these alkaloids have better anti-inflammatory activity than aqueous extracts (Aguilar et al., 2002; Domingues et al., 2011a; Farias et al., 2011; Lozada- Requena et al., 2015; Xu et al., 2021). However, the anti-inflammatory activity of aqueous extracts of *U. tomentosa* is well documented (Roque et al., 2009; Castilhos et al., 2015; Azevedo et al., 2018; Elgawish et al., 2019; Aldayel et al., 2021), and extracts without alkaloids have been shown to maintain their anti-inflammatory activity (Sandoval et al., 2002).

The chemical composition of *U. tomentosa* extracts is often diverse. In addition to alkaloids, the presence of other compounds such as quinovic acid and polyphenols, which contribute to the pharmacological activity of the species, has been reported (Aquino et al., 1991; Yépez et al., 1991; Dietrich et al., 2014). Furthermore, synergism between different compounds usually contributes to the pharmacological effect of medicinal plants (Carmona and Pereira, 2013).

Interestingly, 54% of the studies included in this review used extracts directly related to traditional formulations (aqueous and hydroethanolic extracts). So important is the traditional use that only these studies could be included in the meta-analysis. On the other hand, although 46% of the studies have used extracts not directly related to traditional formulations, they are important because they can help elucidate mechanisms of action (Table 1).

Overall, the studies were at high risk of bias because most of them did not report sample size calculations, the number of excluded animals, or possible conflicts of interest. The main limitation of this study is the small number of studies included in the meta-analysis. Therefore, as more studies are conducted, other pharmacological effects of this species might be demonstrated. Another limitation is the considerable variation in the plant parts used and in the chemical profiles of the extracts, which is a fact that makes interpretation of these results challenging. Nevertheless, as preclinical studies confirmed the anti-inflammatory and/or immunomodulatory effects and the low toxicity of *U. tomentosa* extracts, clinical studies should be encouraged.

## 5 Conclusion

Extracts of the stems, stem barks, roots, and leaves of *U. tomentosa*, mostly aqueous and hydroethanolic extracts, exhibited anti-inflammatory and/or immunomodulatory activities and low toxicity. These extracts decreased NF-κB and the cytokine IL-6 without altering IL-1, IL-10, or TNF-α. These findings suggest that this species has the potential to treat inflammatory diseases associated with increased IL-6 and/or NF-κB, according to the ethnopharmacological use. These activities are not related to a specific class of compounds.

## Data Availability

The raw data supporting the conclusion of this article will be made available by the authors, without undue reservation.
